# Transmembrane protein 25 abrogates monomeric EGFR‐driven STAT3 activation in triple‐negative breast cancer

**DOI:** 10.1002/mco2.492

**Published:** 2024-03-26

**Authors:** Chakrabhavi Dhananjaya Mohan, Kanchugarakoppal S. Rangappa, Gautam Sethi

**Affiliations:** ^1^ FEST Division CSIR‐Indian Institute of Toxicology Research Lucknow Uttar Pradesh India; ^2^ Institution of Excellence, Vijnana Bhavan University of Mysore Mysore Karnataka India; ^3^ Department of Pharmacology Yong Loo Lin School of Medicine National University of Singapore Singapore Singapore

## Abstract

In wild‐type cells, TMEM25 physically associates with EGFR monomer and suppresses the EGFR‐mediated STAT3 phosphorylation, which results in the sequestration of unphosphorylated STAT3 in the cytoplasm. In TMEM^‐/‐^ cells, EGFR monomer phosphorylates STAT3 at the basal level.

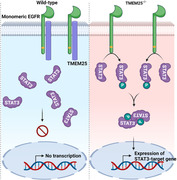

1

In a recent publication in *Nature Communications*, Bi and colleagues demonstrated the importance of TMEM25 (transmembrane protein 25) in the regulation of epidermal growth factor receptor (EGFR)‐dependent STAT3 (signal transducer and activator of transcription 3) activation in triple‐negative breast cancer (TNBC). They proved that lack of TMEM25 enables monomeric EGFR to activate STAT3 in a ligand‐independent fashion in preclinical TNBC models, suggesting the importance of blocking the monomeric EGFR/STAT3 signaling axis in TNBC.^1^ TNBC represents one of the aggressive types of breast cancer, and it accounts for approximately 15% of total breast cancers. The molecular features of TNBC include the lack of expression of estrogen receptor, progesterone receptor, and human epidermal growth factor receptor 2 (HER2). Strikingly, deficiency of these receptors reduces the availability of therapeutic options as they often display unresponsiveness to conventional breast cancer therapeutics, including endocrine therapy (tamoxifen and aromatase inhibitors) and HER2‐targeted therapies (trastuzumab). The discovery of an alternative molecular therapeutic target in TNBC patients is underway in various laboratories across the globe. Concerning this, it is need of the hour to understand the molecular mechanisms that are driving the proliferation and aggression of TNBC to discover effective target‐based therapeutics for the treatment of TNBC patients.

It has been noted from earlier studies that overactivation of EGFR is one of the common events that is witnessed in various types of human cancers. Overexpression of EGFR has also been noted in over 50% of TNBC cases, indicating that EGFR could be the driver of tumor progression. Classically, the binding of EGF to EGFR results in the homo/heterodimerization and subsequent receptor activation to relay the stimulatory signals to downstream EGFR‐dependent pathways such as PI3K/AKT, MAPK, and STAT3 cascades (Figure 1A). These signaling pathways promote cell proliferation, antiapoptosis, angiogenesis, epithelial–mesenchymal transition, metastasis, and therapeutic resistance. Theoretically, the blockade of EGFR must effectively abrogate the proliferation of cancer cells. Conversely, blocking of EGFR using cetuximab (an EGFR‐directed monoclonal antibody) displayed insignificant efficiency in TNBC patients suggesting the dependence of TNBC cells on some other mechanism for their growth. Besides, STAT3 is one of the prominent oncogenic transcription factors that is classically activated by IL‐6 family cytokines. STAT3 is persistently activated in many types of human cancers including TNBC. The interaction of IL‐6 with its corresponding receptor leads to the activation of cytoplasmic nonreceptor tyrosine kinases (such as JAKs and Src) and subsequent phosphorylation of STAT3 on Y705. The phosphorylated STAT3 monomers undergo dimerization, resulting in their nuclear translocation and transcription of genes associated with oncogenic proteins. We have discovered more than 50 inhibitors of STAT3 signaling so far and demonstrated that STAT3 is a good target to block the growth of various cancer cells. Additionally, STAT3 can also be phosphorylated through activated dimeric EGFR to promote oncogenic functions. TMEM family comprises proteins that are poorly described functionally. Some TMEM family proteins are found to modulate the function of the EGFR. Elevated aggressiveness of wild‐type p53 gliomas could be due to the elevation in TMEM167A‐dependent EGFR activity.^2^ TMEM16A was found to regulate EGFR and HER2 in head and neck cancers, and their co‐targeting potently induced cytotoxicity. TMEM25 is a tumor suppressor protein that spans the plasma membrane once, and the precise mechanism of TMEM25 remains elusive. TMEM25 was identified as a favorable prognostic and predictive marker in breast cancer patients.^3^ A previous study has noted a significant methylation of CpG sites (69.2%) in the *TMEM25* gene and the corresponding reduction in its expression in colon tumor tissues in comparison with normal colon mucosa.^4^

**FIGURE 1:**
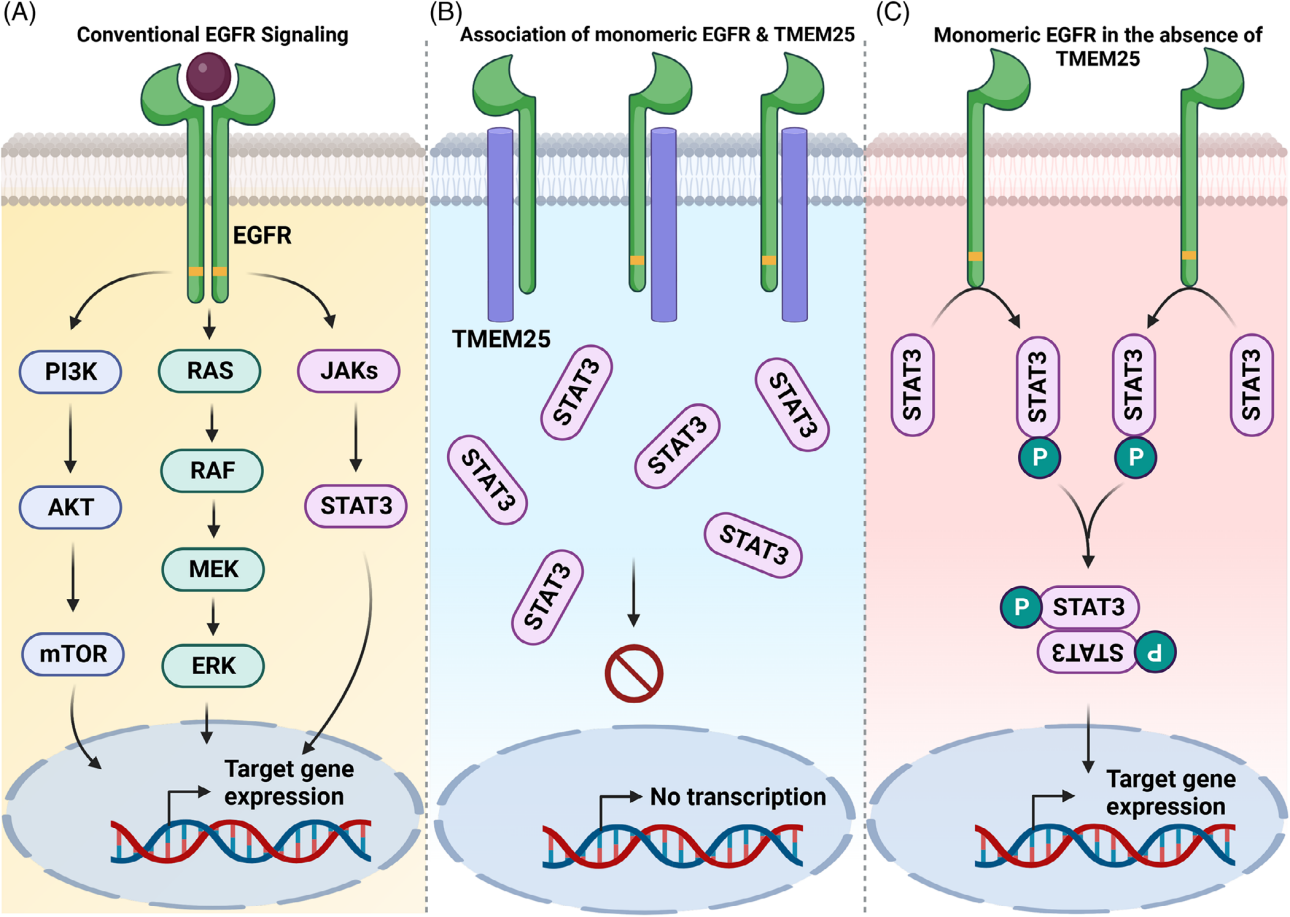
(A) The interaction of EGF with EGFR results in the activation of receptor tyrosine kinase activity, which subsequently leads to the stimulation of EGFR‐dependent signaling pathways including PI3K/AKT, MAPK, and JAK/STAT3 axes. (B) TMEM25 physically associates with EGFR monomer and suppresses the EGFR‐mediated phosphorylation of STAT3, which results in the sequestration of unphosphorylated STAT3 in the cytoplasm. (C) In some cancers (including TNBC), the expression of TMEM25 is downregulated during which EGFR monomer phosphorylates STAT3 at the basal level to drive the activation of STAT3. The dimeric STAT3 shifts into the nucleus to transcribe the target genes which promotes tumor progression.

Bi and colleagues demonstrated the relationship between EGFR, TMEM25, and STAT3 in cell‐based studies and preclinical TNBC models.^1^ They demonstrated the interaction between exogenously expressed or endogenous EGFR and TMEM25 using co‐immunoprecipitation. Using fluorescent techniques, it was shown that EGFR and TMEM25 co‐localize at the plasma membrane. The physical interaction between EGFR and TMEM25 was identified to occur at both carboxy‐terminus (intracellular) as well as amino‐terminus (extracellular) domains of each protein as per the results of domain mapping assay, whereas the in vitro glutathione S‐transferase pull‐down assay detected the interaction between cytoplasmic domains of EGFR and TMEM25. Importantly, the transcript levels of *TMEM25* were significantly low in tumor tissues derived from TNBC patients in comparison with adjacent normal counterparts. Deletion of TMEM25 significantly promoted the growth of TNBC cells in vitro and in vivo, whereas overexpression of TMEM25 displayed opposite effects, suggesting that TMEM25 plays a crucial role in tumor progression. The regulatory role of TMEM25 in the spontaneous development of TNBC was investigated using the knockout system generated by crossing the *TMEM25* ^−/−^ mice with mouse mammary tumor virus LTR‐driven polyoma middle T antigen transgenic mice. Notably, the tumor growth and lung metastasis were evident upon TMEM25 knockout, and overexpression of TMEM25 regressed these effects indicating that TMEM25 displays inhibitory activity on spontaneous TNBC in mice.

The effect of TMEM expression on EGFR‐dependent signaling pathways was examined using *TMEM25^+/+^* and *TMEM25^−/−^* mouse embryonic fibroblast cells. In an interesting observation, phospho‐STAT3 levels were markedly elevated in *TMEM25^−/−^* cells independent of EGF stimulation, suggesting that STAT3 is being activated by some mechanism other than EGFR pathway. On the other hand, forced expression of TMEM25 significantly downregulated EGFR‐driven STAT3 activation, indicating that TMEM25 may play a crucial role in abrogation of convergence of EGFR activation toward STAT3 phosphorylation. It is also important to note that deletion of TMEM25 led to the basal activation of STAT3, while the EGFR itself and all its dependent pathway proteins remained in the unphosphorylated form inferring that basal STAT3 activation is independent of PI3K/AKT and MAPK pathways. The observations were made upon the overexpression or depletion of TMEM25 in TNBC cell lines as well as spontaneous tumors derived from *PyMT* mice (*TMEM25^−/−^* and *TMEM25^wt/tg^*), suggesting the regulatory role of TMEM25 in EGFR/STAT3 axis.

JAKs and Src also mediate the STAT3 phosphorylation, besides EGFR. Depletion of JAK1, JAK2, and Src displayed feeble downregulation, while the depletion of EGFR showed marked suppression of STAT3 phosphorylation in *TMEM25^−/−^* TNBC cells. Also, pharmacological inhibition of EGFR using gefitinib or erlotinib in *TMEM25^−/−^‐*depleted TNBC cells led to the negation of STAT3 phosphorylation, suggesting that EGFR is essential for STAT3 activation in TMEM25‐depleted TNBC cells. Classically, the interaction of EGF with EGFR results in activation of STAT3. However, STAT3 activation was found to be independent of EGF, JAK, and Src, while EGFR inhibition negated STAT3 activation in *TMEM25^−/−^* cells. The effect of monomeric EGFR on STAT3 activation was examined in double‐mutant (*EGFR^−/−^* and *TMEM25^−/−^*) TNBC cells that were transfected with wild‐type EGFR and EGFR^V948R^ vectors. EGFR^V948R^ mutant cannot form a dimer to initiate signal transduction. EGFR^V948R^ transfection retained STAT3 phosphorylation in *EGFR^−/−^*, *TMEM25^−/−^* TNBC cells, strongly suggesting that EGFR can phosphorylate STAT3 in the monomeric form in the absence of TMEM25 and thereby relays sustainable proliferative signals to cancer cells to provide the growth advantage. At last, the expression of TMEM25 protein was found to be significantly low in 23 out of 28 TNBC tissue specimens in comparison with surrounding normal counterparts. In addition, 19 samples out of 23 displayed significantly higher phospho‐STAT3 than adjacent normal tissues, suggesting the inverse relationship between the expression of TMEM25 and phospho‐STAT3 in TNBC. Overall, it is a well‐planned and refined study that comprehensively demonstrated TMEM25 as a tumor suppressor protein that prevents the interaction of monomeric EGFR with STAT3 and thereby suppresses STAT3 phosphorylation and tumor progression (Figure 1B,C).

## AUTHOR CONTRIBUTIONS

C.D.M. wrote the manuscript. K.S.R. and G.S. reviewed and modified the manuscript. All authors read and approved the final version of the manuscript.

## CONFLICT OF INTEREST STATEMENT

The authors declare that they have no conflicts of interest.

## ETHICS STATEMENT

Not applicable.

## FUNDING INFORMATION

Not applicable.

## Data Availability

Not applicable.
